# Differences in Specific Mass Density Between Dinoflagellate Life Stages and Relevance to Accumulation by Hydrodynamic Processes

**DOI:** 10.1111/jpy.13181

**Published:** 2021-06-17

**Authors:** Agneta Persson, Barry C. Smith, Jennifer H. Alix, Yaqin Li, Bridget A. Holohan, Gary H. Wikfors

**Affiliations:** ^1^ Department of Biological and Environmental Sciences Göteborg University Box 461 405 30 Göteborg Sweden; ^2^ National Oceanic and Atmospheric Administration National Marine Fisheries Service Northeast Fisheries Science Center Milford Laboratory 212 Rogers Avenue Milford Connecticut 06460 USA; ^3^ Department of Marine Sciences University of Connecticut 1080 Shennecossett Road Groton Connecticut 06340 USA; ^4^ Smedjebacksvägen 13 SE‐771 90 Ludvika Sweden

**Keywords:** density gradient, dinoflagellate, encystment, gamete, percoll, *Scrippsiella lachrymosa*, sexual life stage

## Abstract

One previously unstudied aspect of differences between sexual and asexual life stages in large‐scale transport and accumulation is density (mass per unit volume) of cells in each life stage. The specific density was determined for *Scrippsiella lachrymosa* cells in medium with and without nitrogen (N) enrichment through density‐gradient centrifugation. Growth medium without N addition is often called “encystment medium” when used for the purpose of resting cyst formation in cyst‐forming dinoflagellates; mating gametes are usually seen after 2–3 days. Significant differences in specific density were found after 2 days in encystment medium simultaneously with the observation of typical gamete swimming behavior and mating. The specific density of cells in encystment medium was 1.06 g · cm^−3^; whereas, the specific density of cells in growth medium was 1.11 g · cm^−3^. Cells in encystment medium were found to have significantly increased lipid content, reduced chlorophyll content, and reduced internal complexity. The findings may explain differential transport of less dense and chemotactically aggregating gametes into surface blooms in contrast to denser vegetative cells that perform daily vertical migration and do not aggregate. Passive accumulation of non‐migrating gametes into layers in stagnant water also can be explained, as well as sinking of zygotes when the storage of highly dense starch increases. Resting cysts had a density of over 1.14 g · cm^−3^ and would sink to become part of the silt fraction of the sediment. We suggest that differences in behavior and buoyancy between sexual and asexual life stages cause differences in cell accumulation, and therefore large‐scale, environmental transport could be directly dependent upon life‐cycle transitions.

**Abbreviations**
FL3: red fluorescence
FSC: forward scatter
SSC: side scatter

Dinoflagellates constitute one of the major phytoplankton groups in both marine and freshwater environments globally. Recent investigations using biomolecular tools have shown that dinoflagellates constitute a large proportion of total protists in all size classes from pico‐ to microplankton (Le Bescot et al. 2016). These organisms are evolutionarily very old, and the phylum occupies different niches and exemplifies vastly different life strategies. Planktonic dinoflagellates constitute an important part of the food web; the majority of species are harmless, providing a valuable food source rich in oil and starch. Nevertheless, some of these single‐celled microscopic organisms are notorious for causing toxic algal blooms worldwide (Anderson et al. 2012). Only about 2% of algal species have been reported to produce harmful blooms, and of these species 75% are dinoflagellates (McLean 2013). Toxigenic dinoflagellates cause many different kinds of poisoning syndromes affecting humans (shellfish poisonings DSP, PSP, NSP, and Ciguatera poisoning), poisonings that cause fish kills (Cusick and Sayler 2013), and toxin bio‐concentration throughout the food web affecting higher trophic levels (Landsbeg et al. 2009).

Many dinoflagellate species, especially in temperate areas (Persson et al. 2000), and notably many of the toxic ones (Faust and Gulledge 2002), have a life cycle including a sexually produced resting stage—the resting cyst. The sexual life cycle involves formation of sexual cells (gametes) and fusion of gametes into a zygote that, after a period of nutrient storage formation, transitions into an immobile resting cyst that falls to the bottom where it spends a resting period. Dinoflagellate resting cysts are known to be able to survive for decades in anoxic sediments (Lundholm et al. 2011, Feifel et al. 2015). It is not unusual for the resting period to be the longest part of the lifecycle; whereas, the growth period and bloom are of short duration (e.g., weeks to one or two months; Dale 1983).

Many factors together contribute to dinoflagellate blooms; not only is growth during favorable conditions important, but also weather patterns, down‐ and upwelling, currents, stratification, and the accumulation of cells by active swimming. Janowitz and Kamykowski (2006) described trapping of cells in the vicinity of a near‐shore front with a biological swimming model driven by chemotaxis. Also, Franks (1997), Durham and Stocker (2012), and Durham et al. (2013) have described hydrodynamic trapping of swimming cells. Different sexual life stages of dinoflagellates have different swimming behaviors (Persson and Smith 2013, Persson et al. 2013), which means that different hydrodynamic trapping can be expected to occur for different life stages. Gametes accumulate, possibly involving positive chemotaxis, and accumulated cells continue to condense into larger patterns (Persson and Smith 2013). This behavior may be one of the most important underlying contributors to the accumulation of dinoflagellate cells known as “blooms.” An assembly of cells close enough to enable sexual reproduction is necessary for the formation of resting cysts, without which survival through extended resting periods (and thus ultimately survival of the species) could not occur. If not interrupted (dispersed) by unfavorable weather patterns or attack by viral infections, parasites, or bacteria, (causing reduced cell proximity) resting cyst formation is a product of a bloom of a cyst‐producing dinoflagellate, promoting a “seed bank” of cysts available for future favorable growth conditions. It is tempting to compare the seed production of annual land plants, and their seed dormancy in the soil, to this “seed bank” of dinoflagellate resting cysts.

The question of whether or not gamete cells have the same specific density as vegetative cells has not been addressed previously. Together with differences in swimming behavior, differences in specific density may explain how different life stages can be caught in and transported by hydrodynamic processes and also accumulate in thin layers in calm water. The study reported here intended to clarify this using a previously well‐studied, cyst‐producing dinoflagellate as a model organism.

*Scrippsiella lachrymosa* is one of several cyst‐producing species of the genus *Scrippsiella*. The species forms resting cysts with an outer wall covered with calcite crystals (e.g., Lewis 1991). *Scrippsiella* species are nontoxic but bloom‐forming, with the most commonly described species of the Genus being *S*. *trochidea* that forms blooms globally (e.g., Ishikawa and Taniguchi 1996). The strain of *S*. *lachrymosa* used here was isolated as a cyst from surface sediments of Casco Bay, Gulf of Maine. It has an exceptionally high cyst‐forming capability (Olli and Anderson 2002) and is therefore an ideal model organism for studies of differences between sexual life stages of dinoflagellates. The common method used for cyst production is transfer to nitrogen‐poor growth medium, commonly called “encystment medium” in studies of cyst formation (e.g., Persson et al. 2008 and references therein). Gametes are known to form within 2‐4 d after transfer to such medium. Two to three d in experiments with *Alexandrium fundyense* and *S. lachrymosa* (Persson and Smith 2013), 2–4 d for *Lingulodinium polyedrum* (Figueroa and Bravo 2005) and also for *Alexandrium tamutum* and *A*. *minutum* (Figueroa et al. 2007).

For *Scrippsiella lachrymosa,* the time for induction is known to be 2–3 d (Persson et al. 2016).

Percoll is a product used for centrifugation in density gradients; it is composed of colloidal silica coated with polyvinyl pyrrolidone. It is harmless to living organisms and is used widely in cell research (GE Healthcare 2007). Percoll can be diluted with salt, sugar, or nutrient solutions to form preparations of different densities that can be layered into tubes without these layers mixing. When adding preparations of different densities beneath a cell culture and then centrifuging this, the cells will sink to a layer that corresponds to their own density. In this way, cell density can be determined, and cells with different densities can be separated from each other. A method for density gradient centrifugation of marine microalgae was developed by C. A. Price (Price et al. 1978, Price 1982). These authors used Percoll and sorbitol to provide a solution with an osmotic potential and density suitable for marine microorganisms. We applied this method to measurements of *Scrippsiella lachrymosa* cultures under experimentally varied conditions.

## Materials and Methods

The project was performed in March 2014 at NOAA/NMFS Milford Laboratory in Milford, CT, USA. Methods development was performed in October 2013 at the University of Connecticut (UCONN) in Avery Point, followed by a pilot experiment at NOAA/NMFS Milford Laboratory (also in October 2013).

For methods development, we had access to cultures of different ages wherein various life stages existed simultaneously. A density gradient centrifugation method was developed that yielded two distinct layers of a *Scrippsiella lachrymosa* culture that contained both vegetative cells and gametes. Two sharp bands were formed at densities 1.06 and 1.11 g · cm^−3^. The upper band contained cells that were slightly smaller (˜17 µm long protoplast) and rounder than cells from the lower band, which were clearly drop shaped and larger (˜20 µm long protoplast). The cells in the upper band were interpreted to be gametes and those in the lower layer were interpreted to be vegetative cells based upon their swimming behavior (circular motion with contact seeking for gametes and straight swimming paths without cell contact for vegetative cells; Persson and Smith 2013, Persson et al. 2013). This led to the design of a pilot experiment with the objective to study in detail the formation of gametes, which typically occurs within 2–4 days after the cells are placed in culture medium without nitrogen (Persson et al. 2008 and references therein). This first experiment was set up to study the initial time period, 0‐4 d, in a 12:12 h L:D lighted bio‐incubator, 20°C, and 222 μE · m^−2^ · s^−1^, with cells in synchronized cell division. The results revealed differences in density of cells between encystment and growth medium starting on day 3 and being clearly evident on day 4 when the experiment ended. There were also differences between morning and afternoon samples in size and chlorophyll *a* content.

A subsequent experiment, reported in detail, was replicated, covered a longer time period, and used 24 h light enabling the study of cultures with unsynchronized cell division. In this way, differences attributable to time of day were eliminated and cells representing a cross‐section of existing stages and phases of division and fusion could be studied and compared between treatments. The use of night and day would be more natural as *Scrippsiella lachrymosa* divide only at night in synchronized cultures; however, choosing the exact and best time for sampling could be difficult, and *S*. *lachrymosa* grow well in 24 h light. The setup, materials, centrifugation, and analysis were otherwise identical between the two experiments, and the pilot experiment is referred to briefly in the Results and Discussion sections.

### Culturing

A culture of *Scrippsiella lachrymosa* (strain B‐10 from Woods Hole Oceanographic Institution, identical to CCMP 2666) in exponential growth was concentrated aseptically by removal of old medium with a pipette covered with a piece of sterile, 10‐µm plankton screen. A 4‐mL aliquot of this original culture was added to each of 80 test tubes (50‐mL glass test tubes) containing 36 mL of medium based upon Milford Harbor seawater. Half (40 tubes) contained f/2 enrichment (Guillard and Ryther 1962) and half contained “Encystment medium”; f/2‐ N (without the addition of nitrogen sources). The starting cell count was 11,000 cells · mL^−1^.

The experiment was performed on glass shelves in a culturing room at 17°C with 24 h fluorescent light at 400 μE · m^−2^ · s^−1^. Stock cultures were kept in this room and thus were adapted to these conditions.

### Sampling

Sampling was performed at 13:00 every day by harvesting three tubes from each treatment (f/2 and f/2‐N) and treating each of them in the following way:

1. 1 mL was sampled for flow‐cytometric measurements of size (as forward scatter, FSC), chlorophyll *a* content as red fluorescence (FL3) at 670 nm, internal complexity (as side scatter, SSC), FL1 at 530 nm for measurement of neutral lipid content with BODIPY stain (Molecular Probes), and cell number (from dot plots of FSC and FL3).
2. 20 mL were preserved with 125 µL 50% glutaraldehyde for Coulter counter cell size determination.
3. 8 mL were used for density gradient centrifugation.
4. 5 mL were used for fluorescence measurements.

### Density gradient centrifugation

The method developed by C.A. Price was used for density gradient centrifugation; Percoll sorbitol preparations with magnesium TRIS seawater (Price 1982, p 266) of different densities were tested during methods development in October 2013 until preparations having densities yielding good separation of different life stages into two distinct layers were found.

Stock solutions: “10X Mg‐TRIS‐Seawater”: 15.25 g MgCl·6H_2_O, 20.15 g TRIS base, and 13.2 g TRIS‐HCl was diluted to 500 mL with seawater. “1.11X Sorbitol”: 25.2 g sorbitol was diluted into 250 mL in distilled water. “1.11X Percoll‐Sorbitol”: 25.3 g sorbitol was diluted into 250 mL Percoll. From these solutions the “Starting solution” (90 mL 1.11X Sorbitol and 10 mL 10X Mg‐TRIS‐Sw) and “Final solution” (90 mL 1.11X Percoll‐Sorbitol and 10 mL 10XMg‐TRIS‐Sw) were made by mixing in different proportions to create preparations of desired density. Replicate samples of all solutions were measured in calibrated pipettes and weighed on an analytical balance at 20°C. Density marker beads (Amersham Biosciences Density Marker Beads, Pharmacia Biotech, Uppsala, Sweden) for calibration of Percoll gradients used extra tubes centrifuged simultaneously with those in the experiment.

For density gradient centrifugation, 8 mL culture was placed in a 15 mL polypropylene centrifuge tube, and the Percoll preparations with different densities were placed carefully under the culture with long Pasteur pipettes that had been calibrated to 1.5 mL (marked at 1.5 mL with a permanent marker). Each layer was placed under the less dense in order; culture; 64.3% final; 66.7% final and 100% final solutions were used having densities of 1.029; 1.103; 1.104 and 1.14 g · cm^−3^ at 20°C, thus creating “cushions” separating cells that were less dense than 1.10 g · cm^−3^ from those denser, but with a lower density than 1.14 g · cm^−3^. Cells denser than 1.14 g · cm^−3^ ended up at the bottom of the centrifuge tube. For another density determination, extra centrifugations with a “self‐generated” gradient were performed according to instructions on Percoll gradients, in GE Healthcare (2007), using density marker beads and extra *Scrippsiella lachrymosa* cultures with different life stages present simultaneously.

Centrifugation was performed for 5 min in a centrifuge (Thermo Electron Multi RF with a #8947 rotor) at 1,000*g* at 16°C. Acceleration and deceleration were slowed to not disturb the layers.

Photography of the resulting layers was performed with ProScope HR, each day in the same place with the same lighting to facilitate comparison of layers over time. Photographs were analyzed with the image‐analysis software program ImageJ (https://imagej.nih.gov/ij/); the “gradient tool” was used to capture a gradient in the photo of each tube, and values were exported to Excel.

### Microscopy of cells of different density

Cells were studied daily in the microscope (Zeiss, Göttingen, Germany, Axioskop 2 mot plus). Cells were studied before and after centrifugation, and cells that accumulated in different layers upon centrifugation were compared and photographed.

### Analysis of cell size

To achieve objective measurements of cell sizes, we used a Coulter counter (Beckman Multisizer 11e, Danvers MA, USA). Also, flow cytometry provided a cell size measurement as FSC; although it was not possible to calibrate FSC with true size, as FSC values are partially dependent upon differences in cell surface texture and configuration in addition to cell size.

### Flow cytometry

A BD BioSciences Facscan flow cytometer (San Jose, CA, USA) was used to measure several cell characteristics. Samples were analyzed live each day (30 s acquisition), starting at 13:35. After measurements of unstained cells, 2 µL of the fluorophore BODIPY (final concentration 5 µM) was added to each sample to analyze neutral lipid content using green fluorescence at 530 nm (FL1; Hyka et al. 2012); samples were placed in the dark 10 min before analysis. Other flow‐cytometric measurements used were: FSC as a measurement of relative size, SSC as a measurement of the internal complexity, and red fluorescence (FL3) at 670 nm as a measurement of chlorophyll *a* content.

### Measurement of variation in chlorophyll fluorescence

The FIRe (Fluorescence Induction and Relaxation) System (Satlantic Inc., Halifax, Canada) was used for variable chlorophyll fluorescence measurements. FIRe is based upon the Fast Repetition Rate Fluorometry (FRRF) technique. Algal culture samples were kept in the dark at ambient water temperature for at least 20 min before measurements. *F*_v_/*F*_m,_ maximum quantum efficiency or maximum quantum yield of photosystem II (PSII), was calculated through the built‐in instrument protocol.

### Statistics

For each dependent variable and point in time, comparisons between the life stages were made using one‐way ANOVA (StatGraphics Plus 5.1., Statpoint Technologies, Inc., Warrenton, VA, USA). The method used to discriminate between means was Fisher’s least significant difference procedure at the 95% level (*P* < 0.05). Error bars in figures represent standard deviation.

## Results

### Density gradient centrifugation and density determination

**Fig. 1:**
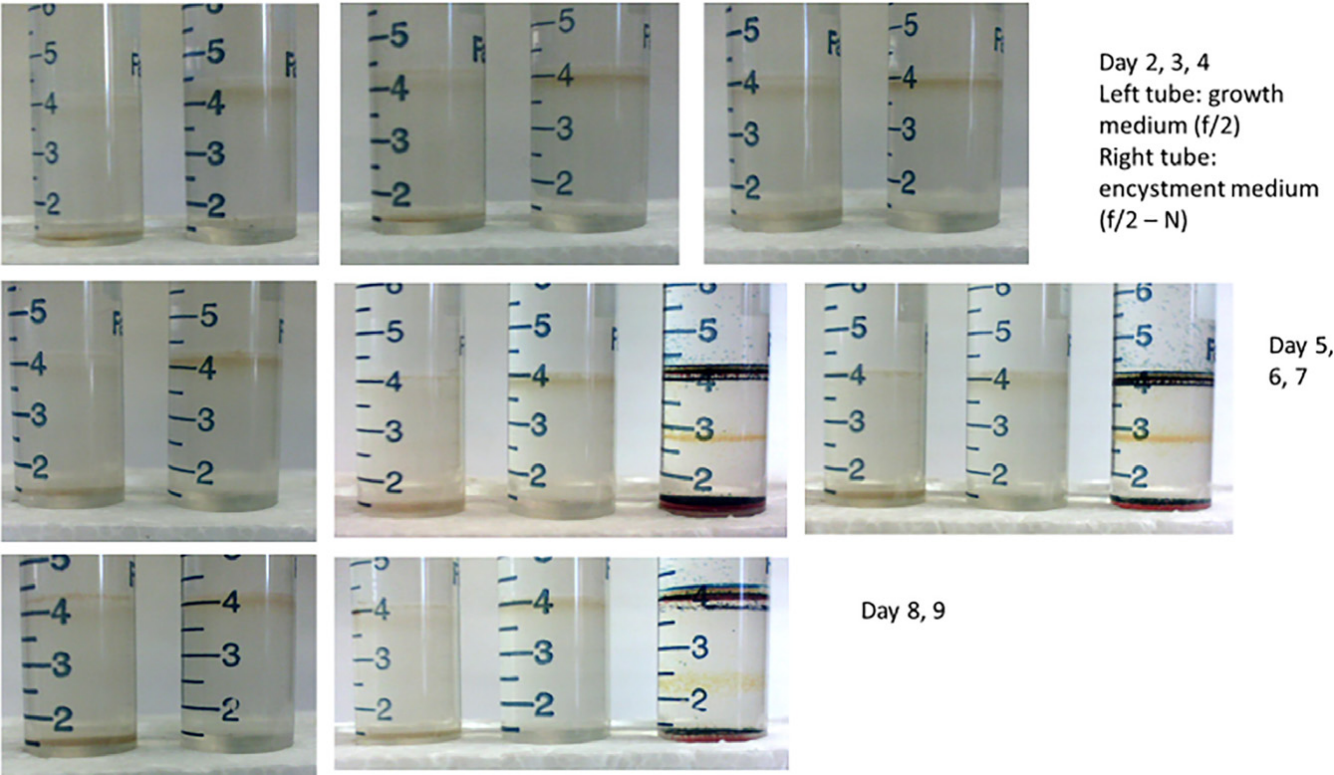
Photographs from different points in time showing large and consistent differences in density between cells in f/2 growth medium (vegetative cells) and cells in encystment medium (putative gametes). The third tube of day 6, 7, and 9 have density marker beads. [Color figure can be viewed at wileyonlinelibrary.com]

**Fig. 2:**
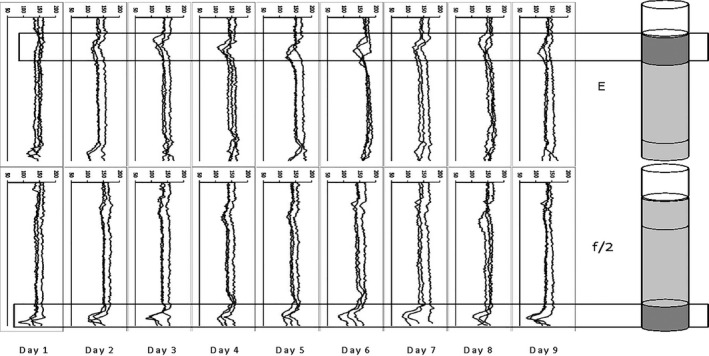
Density gradient centrifugation. The photographs are summarized by showing gray scale transects from photographs of each tube (three replicates per treatment and day) where low values represent darker color (more cells). “E” are cells from encystment medium and “f/2” are cells from growth medium.

Differences between treatments appeared very fast. First (day 1), cells in both treatments formed a band on top of the densest Percoll layer, but by day 2, cells in encystment medium were forming a band on top of the less dense Percoll layer, while cells in growth medium were not. This difference persisted and increased throughout the experiment (Fig. 1), showing large differences in density between cells in f/2 growth medium (vegetative cells) and cells in encystment medium (putative gametes). Figure 2 summarizes the photos by showing gray scale transects from photos of each tube (three replicates per treatment and day) in which low values represent darker color (more cells).

Densities for the respective layers where cells formed bands were determined using density marker beads to be 1.06 g · cm^−3^ for the “gamete” layer, 1.11 g · cm^−3^ for the “vegetative cell” layer and >1.14 g · cm^−3^ for resting cysts that passed through all layers and formed a pellet in the bottom of centrifuge tubes. Resting cysts were formed only in encystment medium, not in growth medium.

### Coulter counter cell size

**Fig. 3:**
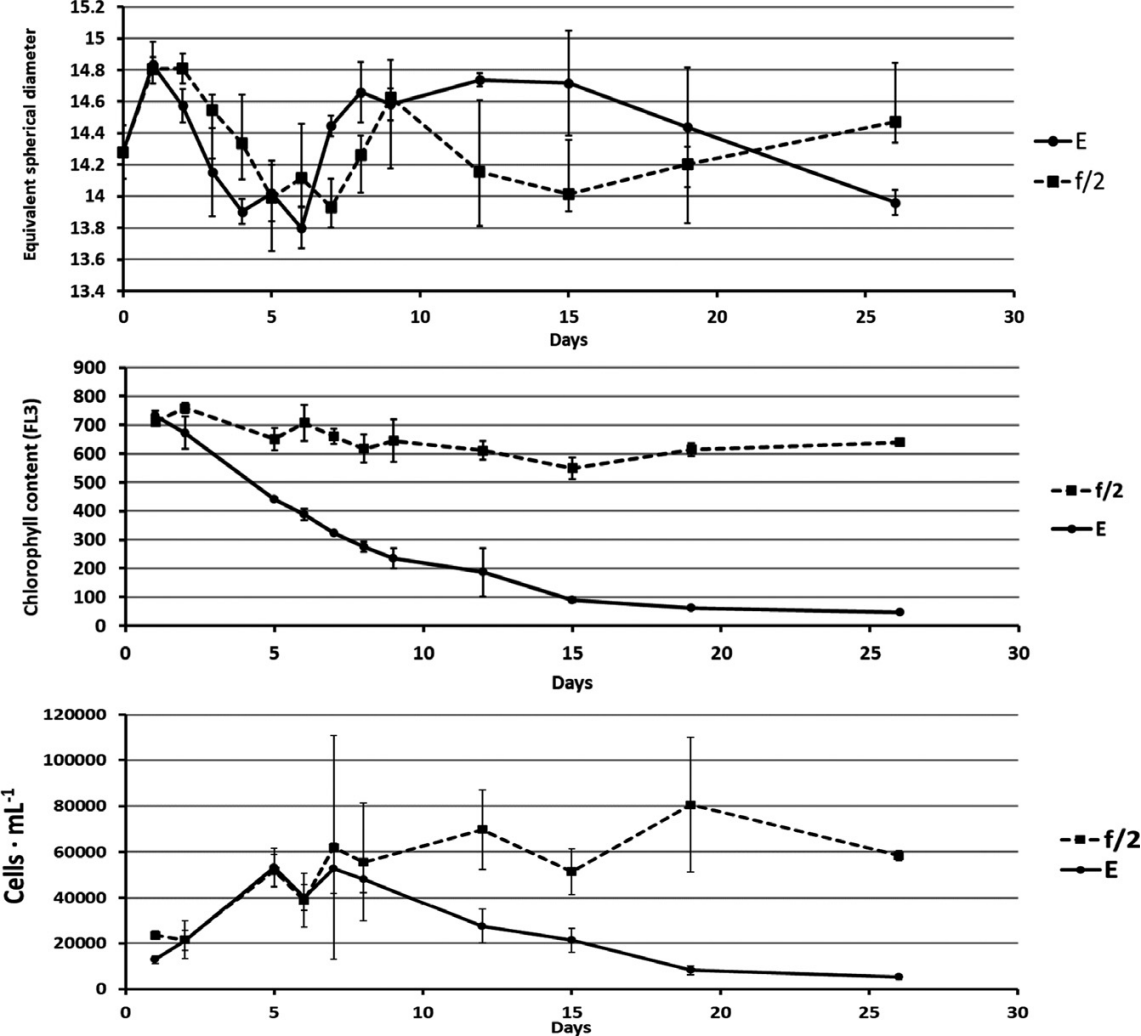
Top: Coulter counter cell sizes of cells grown in f/2 and encystment medium (E), respectively. Middle: Chlorophyll *a* content as red fluorescence (FL3) of cells grown in f/2 and encystment medium (E), respectively. Bottom: Cell numbers of cells grown in f/2 and encystment medium (E), respectively.

The Coulter data for cell size showed significantly decreased average size for cells in encystment medium compared to cells in growth medium during the first part of the experiment. In both treatments, cell size varied significantly over time (Fig. 3). The smallest cells had an equivalent spherical diameter only 7% smaller than the largest. The variation in size increased with time in both treatments, showing an increasing presence of differently sized cells.

### Flow cytometry

#### Forward scatter “cell size”

Initially, the average FSC was higher in the encystment treatment, but during days 5–9, cells in encystment medium were somewhat smaller compared to those in growth medium. There was a discrepancy between FSC and Coulter counter cell size measurements (see above and discussion), and the lowest FSC values were 63% of the highest. Differences in FSC were larger within treatments over time than between treatments.

#### FL3 chlorophyll *a* content

In the preliminary experiment with synchronized cell division and 12:12 h L:D light period, chlorophyll *a* increased during daytime and decreased during the night. In the experiment with 24‐h light and one sampling per day, such day‐night cycling is not detectable. A very large difference between treatments was evident, however, starting the second day (Fig. 3). Cells in encystment medium had significantly reduced chlorophyll *a* content; whereas, cells in growth medium maintained relatively constant chl *a* content.

#### Cell number

Cell numbers initially increased in both treatments, but soon dropped in encystment medium; whereas, cell numbers in growth medium remained high (Fig. 3). The experiment was started with high cell numbers that did not permit continued exponential growth.

#### Internal complexity as SSC

**Fig. 4:**
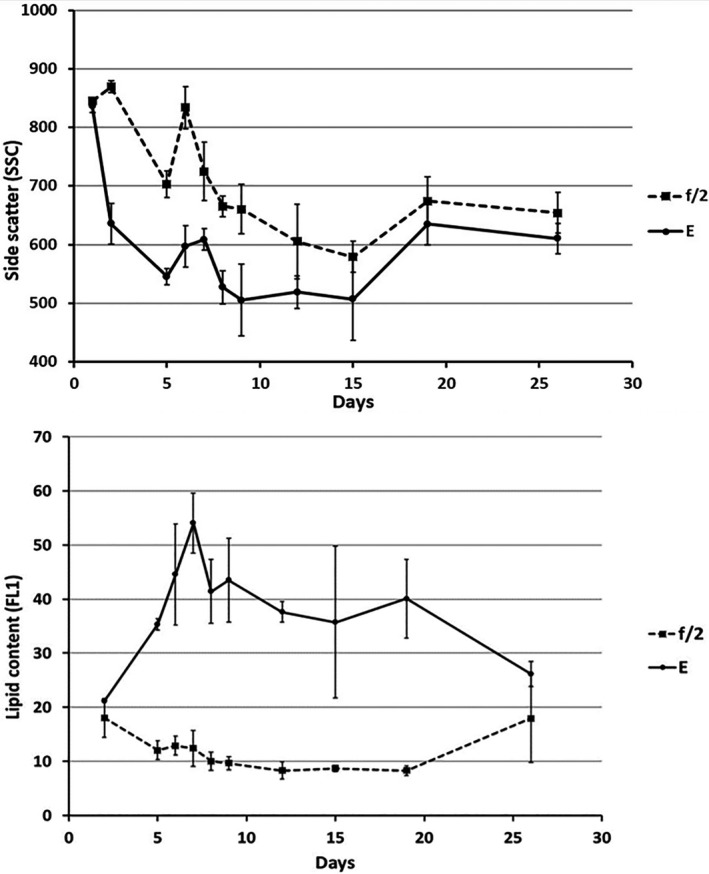
Top: Internal complexity as side scatter (SSC) of cells grown in f/2 and encystment medium (E), respectively. Bottom: Neutral lipid content measured as green fluorescence (FL1) of BODIPY colored cells grown in f/2 and encystment medium (E), respectively.

The internal complexity described by the SSC measurements revealed significant differences between treatments. Cells in encystment medium had a significantly lower internal complexity than cells in growth medium (Fig. 4).

#### Lipid content—FL1 BODIPY

There was a large difference in lipid content between cells in growth medium (“vegetative cells”) and in encystment medium (“gametes”). The putative gametes contained significantly more neutral lipids compared to vegetative cells (Fig. 4).

### Fluorescence

The quantum efficiency of Photosystem II (*F*_v_/*F*_m_) was similar in growth medium and encystment medium. Values decreased after 5 days in both treatments.

### Microscopic observation of cell morphology, lipid droplets, and starch grains

**Fig. 5:**
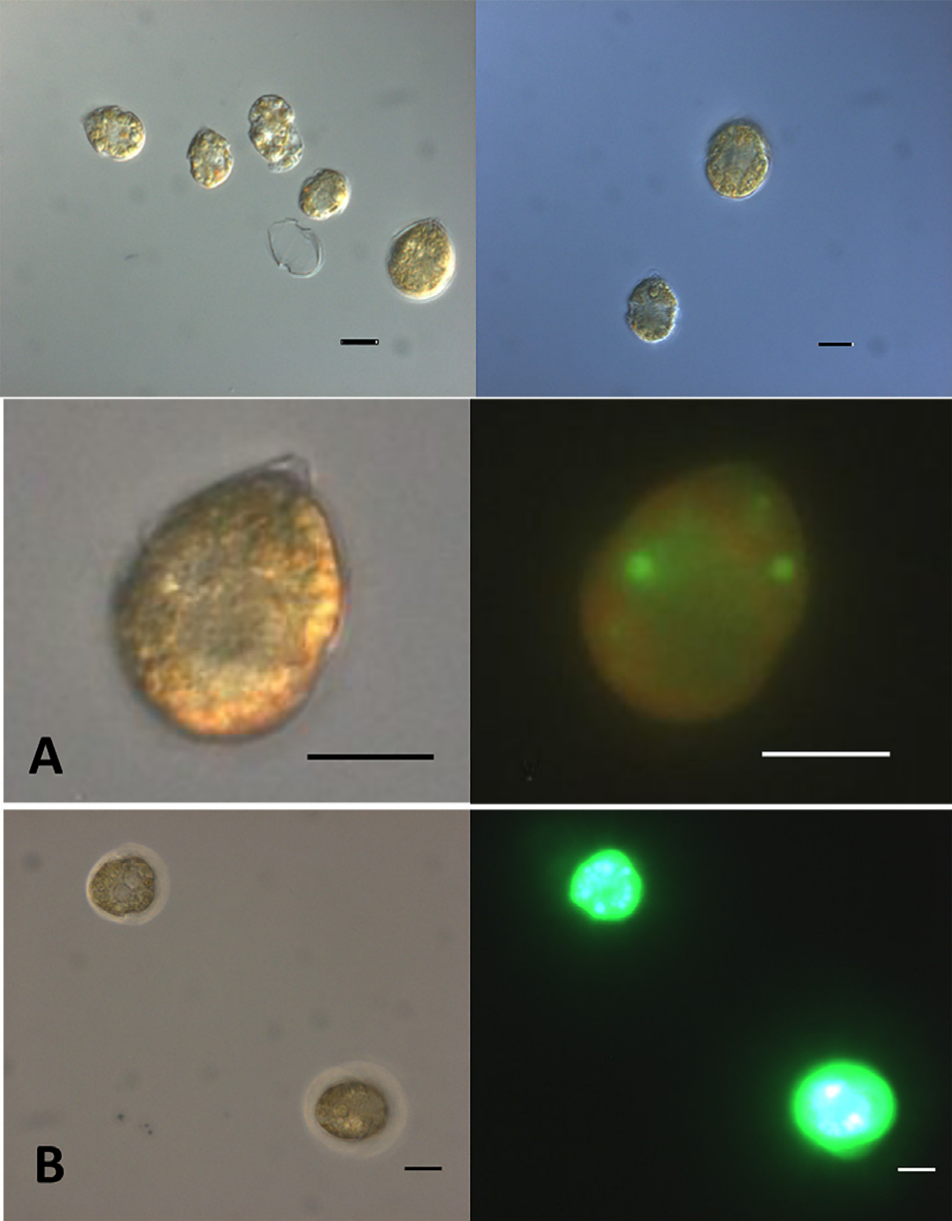
Top Bar: Cells at day 4, within each treatment differently sized cells were present. Left: Encystment medium, putative gametes were present as well as vegetative cells. Right: Growth medium, vegetative cells in unsynchronized growth. (A) Cell in f/2 growth medium on day 5 stained with BODIPY. (B) Cells in encystment medium on day 5 stained with BODIPY. Scale bars are 10 µm. [Color figure can be viewed at wileyonlinelibrary.com]

Throughout the experiment, the upper band in density gradient centrifuged cultures contained cells that were perceived as somewhat smaller, paler and rounder than cells from the lower band, which were more clearly drop shaped, deeply pigmented and larger. Size differences between cells within the same treatment are shown in Figure 5. As in earlier studies, the cells believed to be gametes swam in a contact‐seeking manner (described for *Alexandrium fundyense* in Persson et al. 2013); whereas, the drop‐shaped vegetative cells swam in straight paths without hitting other cells.

Lipid droplets were clearly visible in cells in the upper band and could also be viewed as brightly green in fluorescence microscopy when stained with BODIPY (Fig. 5, A and B).

**Fig. 6:**
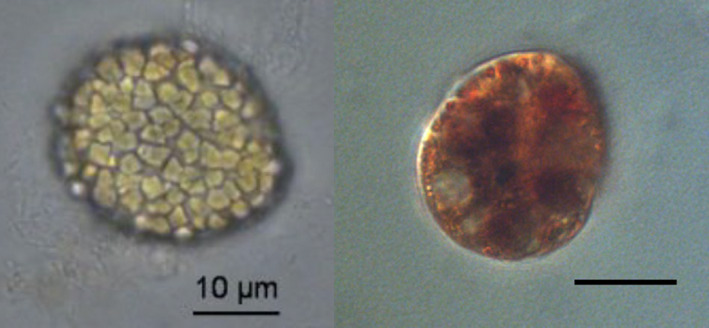
Left: Resting cyst from encystment medium. Right: Lugol‐stained cell. Scale bars are 10 µm. [Color figure can be viewed at wileyonlinelibrary.com]

As cultures were unsynchronized, dividing vegetative cells and zygotes both were present during daytime. Large cells were most often found in the lower band and without large lipid droplets. Mating gametes were observed in the lower layer, not the top layer. Resting cysts (Fig. 6) ended up at the bottom of tubes in density gradient centrifugation (>1.14 g · cm^−3^) and were present in encystment medium from day seven and onwards, but not seen at all in f/2.

It was apparent from microscopic examination of cells from different layers and from the bottom of tubes from density gradient centrifugation that the presence of starch granules contributed to a markedly increased density of the cells. Cells with many starch grains were always found at high‐density bands, never at low. Starch was not quantified in this experiment, but starch grains were visible as irregular granules in the cytoplasm. They were colored dark when Lugol’s iodine was added (Fig. 6).

## Discussion

The present study demonstrated differences in mass per unit volume in vegetative cells, putative gametes, and zygotes of the cyst‐producing dinoflagellate *Scrippsiella lachrymosa*. It appears likely that these differences, affecting buoyancy and sinking, contribute to environmental distribution of different life‐history stages of meroplanktonic dinoflagellates with planktonic growth and reproductive stages and a benthic resting stage. The dinoflagellate bloom paradigm focusing on vegetative growth as the primary driver deserves reconsideration based upon accumulating evidence that life cycle, behavior, and hydrodynamics may have large roles in accumulations of cells in the environment. In 2002, Smayda wrote: “Dinoflagellates seemingly combine the swarming behavior of insects with the classical bloom behavior of phytoplankton in their ecology and adaptive strategies.” Dinoflagellate blooms can appear suddenly with cell numbers not possible to explain by fast growth and in waters without sufficient nutrients to support the number of cells present (Smayda 2010, Smayda and Trainer 2010). Prediction is difficult, and HAB forecasts suffer from the same kinds of inherent environmental uncertainties as weather forecasts, that limit both precision and accuracy. Large efforts are made to construct models (e.g., Anderson et al. 2014, 2014; *Alexandrium fundyense* in the Gulf of Maine) and follow the development of blooms (e.g., *Karenia brevis* in the Gulf of Mexico; https://tidesandcurrents.noaa.gov/hab/gomx.html). Of special interest to the study presented here, McGillicuddy et al. (2014) saw that the clearest connection between *A. fundyense* blooms and any environmental variable was with nitrogen deficiency; *A*. *fundyense* was most abundant where nitrate was most depleted. Nitrogen deficiency is intimately connected with gamete formation but hinders vegetative growth.

Cell density—mass per unit volume—together with shape, size, surface structure (and projections), determines how microorganisms float or sink in a given water mass if they are immobile (Price et al. 1978, Lavoie et al. 1986). Sinking velocity has been studied for many phytoplankton species (Eppley et al. 1967, Bienfang and Harrison 1984, Walsby and Holland 2006), and also for dinoflagellate resting cysts (Anderson et al. 1985). Dinoflagellates in general are highly mobile; accordingly, their specific mass density has not received much attention. Vegetative cells of phytoplankton occupy a broad range of densities from 1.03 to 1.20 g · cm^−3^, with most species that are not heavily silicified or calcareous near 1.05 g · cm^−3^ (Eppley et al. 1967). Van Ierland and Peperzak (1983) determined the density of *Peridinium* sp. to be 1.08 ‐ 1.12 g · cm^−3^. Sinking also depends upon the properties and movement of the water mass (temperature, salinity, stratification, viscosity, velocity, turbulence, etc.). The density of surface seawater ranges from about 1.02 to 1.03 g · cm^−3^, depending upon the temperature and salinity. Deep in the ocean, under high pressure, seawater can reach a density of 1.05 g · cm^−3^ or higher (Nayar et al. 2016). Stratification and separation of water bodies with different properties from each other are well‐known phenomena that act as barriers to water mixing. Motile microorganisms can adjust position using various tactic responses: they can, for example, perform daily vertical migrations, stay at a saturated light level, move to avoid predators or unfavorable environments, or assemble for sexual reproduction. The possibilities dinoflagellates have, however, for determining position are severely restricted by water movements; because of small size, cells live in an environment of low Reynolds numbers and are trapped in and transported by the water when it moves (e.g., Margalef 1997). Studies on trapping of motile phytoplankton (e.g., Janowitz and Kamykowski 2006, Durham and Stocker 2012, Durham et al. 2013) have pointed out the importance of cell motility for the trapping outcome. Thus, when different life stages of the same species have different swimming behaviors and different specific mass density, they may end up in separate water masses, causing them to accumulate in very different ways and be found in different places following differential trapping and transport. From a widespread background population of low cell numbers in a large volume of water, a concentrated surface or layer bloom can appear if cells change specific mass density as well as swimming patterns and accumulate by chemotactic behavior (as they do when the sexual cycle is induced in dinoflagellates).

Experiments reported here show that the density of the bloom‐forming dinoflagellate *Scrippsiella lachrymosa* is significantly different between life stages. Specific mass density was significantly reduced, and lipid formation was significantly increased in nitrogen‐deficient medium. Lipids have a low density; therefore, accumulation causes a reduction in the specific mass density. Also, Fuentes‐Grünewald et al. (2012; *Alexandrium minutum*) and De la Jara et al. (2003; *Crypthecodinium cohnii*) noted enhanced lipid production in dinoflagellates upon N‐starvation. The use of N‐starvation for induction of the sexual cycle is commonplace in dinoflagellate research, and nutrient media without nitrogen addition often are termed “encystment medium” (Persson et al. 2008 and references therein). It is tempting to interpret published results of N‐starvation treatments on dinoflagellates with a known sexual life cycle as describing gametogenesis, but this must be done with caution when no observations of behavior are reported. Studies comparing cells in growth medium with cells in N‐deficient medium may very well be comparing vegetative cells with gametes. Brosnahan et al. (2014) suggested an important role for aggregation at the sea‐surface during sexual events for *Alexandrium* *fundyense*. It is possible that gametes of many dinoflagellate species accumulate large amounts of lipids and that this contributes to observed surface blooms. Having a lower density allow gametes to more easily stay afloat in the water column, thereby acquiring greater opportunity to meet mates.

Clearly, nitrogen deficiency did not induce a classical plant stress response of reduced F_v_/F_m_ (e.g., Warner et al. 1999) in *Scrippsiella lachrymosa*. Putative gametes did not have lower *F*_v_/*F*_m_ compared to vegetative cells (rather somewhat higher). Jauzen and Erdner (2013) and Qi et al. (2013) reported that *F*_v_/*F*_m_ did not change for dinoflagellates under N stress. Qi et al. (2013) concluded that *F*_v_/*F*_m_ cannot be used for detection of N or P stress in *Scrippsiella trochoidea,* and that this method has limitations in the detection of nutrient stress in phytoplankton. In addition to cultured phytoplankton, there were also exceptions in a natural phytoplankton community studied; the population maintained the same level of *F*_v_/*F*_m_ under low nutrient conditions (Li et al. 2015). Parkhill et al. (2001) suggested that some phytoplankton species have the same level of *F*_v_/*F*_m_ under low nutrient conditions when they are under balanced growth condition or acclimated to N‐limitation. We interpret the lack of stress response as further proof that N‐starvation is an important signal for the induction of the sexual cycle, causing a change in metabolism directed at producing reproductive stages instead of asexually dividing cells. This change is not harmful; it is different from a classical or harmful stress response. Putative gametes had lower chlorophyll than the vegetative cells but similar *F*_v_/*F*_m_, which means the two cell types likely had similar photosynthetic capacity per unit chlorophyll *a*.

Another well‐known effect of N‐starvation is starch formation (e.g., Dagenais Bellefeuille et al. 2014). We saw, but did not quantify, increased starch formation, and it was obvious using microscopy of density gradient centrifuged cultures that cells with starch accumulation became more‐dense. The density of a cell is determined by its inner components and the material of its cell wall. There is no such thing as reference density of a cell; density is not constant over time but changes over the cell’s life‐span: every day it changes when oil, chlorophyll, and starch (and other substances) are produced during the light period and consumed at night. Starch has a very high density (1.5 g · cm^−3^; Buléon et al. 1998) and is formed at daytime during photosynthesis and consumed during the night (Behrmann and Hardeland 1995). In N‐starved cells, however, starch is accumulated and not completely consumed at night (Dagenais Bellefeuille et al. 2014). Lipids have a low density (˜0.9 g · cm^−3^; Noureddini et al. 1992) and their formation is enhanced upon N‐starvation (as discussed above). Chloroplasts have high density (˜ 1.22 g · cm^−3^; Miflin and Beevers 1974); in the absence of nitrogen, chlorophyll can no longer be formed, but neither starch nor lipids contain nitrogen and continue to form. Furthermore, starch and lipids are very important storage nutrients necessary for the survival of the resting stage. The nucleus is very dense (˜1.7 g · cm^−3^ for DNA; Ingle et al. 1975), mitochondria have a density of ˜1.15 g · cm^−3^ (Miflin and Beevers 1974), and proteins ˜1.22‐1.35 g · cm^−3^ (Quillin and Matthews 2000). A cell is in constant development; a vegetative cell is always on its way from newly divided to ready to divide again with double contents. Everything that is produced within the cell affects its density and thus how it is transported or settles. Also, the formation of the cyst wall contributes to increased density. Calcareous crystals cover the cysts in the genus *Scrippsiella* (Lewis 1991); whereas, many other dinoflagellate species have a thick wall of sporopollenin (e.g., Dale 1983). Zinssmesiter et al. (2013) described how the calcareous crystals for the resting cyst wall were formed within vacuoles in calcareous dinophyte strains.

The cells thought to be gametes had the lowest density in our study, but as soon as they started the mating process, they became denser. The mating cells transform into a zygote; two cells with all contents combine into one dense cell that eventually will become a very dense resting cyst covered with calcite crystals. The zygote increases in density further by gradually building up polysaccharide storage in the form of starch granules in the cytoplasm. Zygotes often are so tightly packed with both lipid globules and starch granules that they appear darker in the microscope compared to other life stages without having a higher pigment content (Persson et al. 2016).

In this experiment, cells in encystment medium (thought to be mostly gametes) had significantly lower SSC than cells in vegetative growth, indicating a lower number of internal, light‐scattering organelles and/or storage products. Gamete formation in the field may be induced differently than under laboratory conditions, but is always accompanied by cell accumulation (by necessity, otherwise fusion and cyst formation could not take place), which then suggests that nutrient limitation may result from the large number of cells in a limited space. It is common that gametes are perceived as somewhat smaller and more lightly colored (fewer organelles, less storage and less chlorophyll), and this makes it tempting to generalize from *Scrippsiella lachrymosa* to more dinoflagellate species. Cell size alone is not a good indicator of life stage, even though gametes often appear to be somewhat smaller than vegetative cells and in *S*. *lachrymosa* lack the typical drop shape. There was a discrepancy between size measurements with the Coulter counter vs. flow cytometry FSC. The Coulter counter size is a more accurate size measurement as the measured signal is proportional to the volume of the cell. Coulter counter cell size results were in accordance with microscopy, showing the slightly smaller size of gametes until pairs and zygotes started to form and make the average cell size in encystment medium larger again. When differently sized life stages were present, as seen microscopically, the Coulter counter size had a larger variation. Flow cytometry FSC is proportional to the cell‐surface area, but cell shape and surface topography also contribute significantly to the light scatter (BD Biosciences 2000). Even if the slight cell size difference between vegetative cells and gametes is discernable, each time life stages are compared in the microscope (and with the different instruments), it is important to notice that the actual size of cells differs much more over time than between life stages. It is well known that starved or seasonal forms of dinoflagellates can be very small in nature (Silva and Faust 1995). These do not have to be gametes, although sometimes they are. Few, if any, conclusions regarding life stage can be drawn from cell size information alone. Observations of swimming behavior with intense contact seeking and the presence of mating cells are a much more reliable marker of the presence of sexual cells.

Previously, we have shown that *Scrippsiella lachrymosa* forms gametes in encystment medium even when the water is mixed (Smith and Persson 2005). The gametes assemble as soon as the water stops moving. This suggests that gametes could be formed in mixed water masses as a response to seasonal signals, but are unable to accumulate until the water becomes more settled. When the opportunity comes—the water becomes still enough to allow accumulation—gametes accumulate and fuse. This can answer the common question about how dinoflagellate blooms can develop so quickly (e.g., Smayda 2010); if the gametes are ready, when the appropriate conditions that allow assembly and mating occur, they can assemble quickly.

The range in density studied here was optimized for the density gradient differentiation between gametes and vegetative cells in one particular species. For use of the method on other species, specific density ranges first must be found empirically. As an example, *Alexandrium fundyense* was also studied in the pilot experiment, and it had cells of lower density compared to *Scrippsiella lachrymosa*, in the range between 1.05 and 1.10 g · cm^−3^ (with resting cysts >1.14 g · cm^−3^).

An ultimate interpretation of the modest results in the present study contributes to the solution to Hutchinson’s “paradox of the plankton,” in that additional explanations are suggested for the patchiness observed in microbial distribution in the environment. In many ways, nature sorts different species from each other, explaining how they can be found in different places or different layers in the same area.

## Conclusions

Cells of the dinoflagellate *Scrippsiella lachrymosa,* when in encystment medium (putative gametes), have significantly lower specific mass density compared to vegetative cells, caused by a high lipid content, low chlorophyll content, and reduced internal complexity.

Differences in behavior and buoyancy between sexual and asexual life stages may be sufficient to contribute to differences in cell accumulation and large‐scale transport. Environmental factors that cause dispersion for vegetative cells can cause assembly for gametes. This provides a scenario explaining the sudden appearance of dinoflagellate blooms in nature. A widespread growth of vegetative dinoflagellate cells in low numbers can remain un‐noticed, but gametes form upon seasonal signals and then accumulate into large patterns by the synergistic effects of their swimming behavior, specific mass density, and ability to use the water density discontinuities and currents for accumulation.

We are grateful to the reviewers and the editor for constructive comments on earlier versions of the manuscript. Dr. Sandra Shumway not only allowed A.P. to work in her laboratory during the government shutdown in October 2013 (that closed the Milford laboratory for the first week of the research trip), but also let her stay in her house while doing research at University of Connecticut, Avery Point. For this, A.P. is very grateful. Financial support was provided by Ångpanneföreningens forkningsstiftelse and J. Gust. Richerts foundation. Mention of trade names does not imply endorsement.
